# Sex Differences in Post-exercise Hypotension, Ambulatory Blood Pressure Variability, and Endothelial Function After a Power Training Session in Older Adults

**DOI:** 10.3389/fphys.2021.657373

**Published:** 2021-07-16

**Authors:** Leandro de Oliveira Carpes, Lucas Betti Domingues, Renato Schimitt, Sandra C. Fuchs, Taha Alhalimi, Hirofumi Tanaka, Rodrigo Ferrari

**Affiliations:** ^1^Postgraduate Program in Cardiology, School of Medicine, Universidade Federal do Rio Grande do Sul, Porto Alegre, Brazil; ^2^Sports and Exercise Training Study Group, Hospital de Clínicas de Porto Alegre, Porto Alegre, Brazil; ^3^Department of Kinesiology and Health Education, The University of Texas at Austin, Austin, TX, United States

**Keywords:** aging, high blood pressure, blood pressure variability, post exercise hypotension, flow-mediated dilatation, high-velocity resistance training

## Abstract

**Background**: The efficacy of power training (PT) to acutely reduce blood pressure (BP) in participants with hypertension is controversial, and no studies have assessed the influence of sex on post-exercise hypotension and its mechanisms in older adults.

Purpose: The aims of this secondary, exploratory analysis were to compare the effects of a single bout of PT on post-exercise hypotension, BP variability, and endothelial function between older men and women with hypertension.

**Methods:** Twenty-four participants with hypertension (12 men and 12 women aged to >60 years old) took part in this crossover study and randomly performed two experimental sessions: power exercise training (PT) and non-exercising control session (Con). The PT protocol was composed of 3 sets of 8–10 repetitions of five exercises performed in the following order: leg press, bench press, knee extension, upright row, and knee flexion, using an intensity corresponding to 50% of one repetition maximal test (1RM) and 2-min intervals between sets and exercises. The concentric phase of exercises during each repetition was performed “as fast as possible,” while the eccentric phase lasted 1 to 2 s. During Con, the participants remained at seated rest on the same exercise machines, but without any exercise. Each protocol lasted 40 min. Office BP, flow-mediated dilatation (FMD), 24-h ambulatory BP, and the average real variability (ARV) of systolic and diastolic BP were assessed before and after experimental sessions.

**Results:** Comparing PT with Con, a reduced office BP after exercise was found in men (systolic BP—average post 1 h: −14 mmHg, *p* < 0.001; diastolic BP—average post 1 h: −8 mmHg, p < 0.001) and only a reduced systolic BP in women (average post 1 h: −7 mmHg, *p* = 0.04). Comparing men and women, a reduced systolic BP (post 60': −15 mmHg, *p* = 0.048; average post 1 h: −7 mmHg, *p* = 0.046) and diastolic BP (post 60': −9 mmHg, *p* = 0.049) after the first hour were found in men. In relation to 24-h ambulatory BP, ARV, and FMD, no statistically significant differences were found between men and women.

**Conclusion:** In older adults with hypertension, the office BP response after the experimental sessions was different in men and women, showing that the PT protocol is more effective to acutely reduce BP in men. Additionally, the mechanisms behind this reduction remain unclear. This finding suggests that sex cannot be combined to analyze post-exercise hypotension.

Clinical Trial Registration: ClinicalTrials.gov, Identifier: NCT03615625.

## Introduction

Resistance training is a cornerstone intervention to counteract age-related declines in physical function (Peterson et al., 2010), improving independence, and reducing the risk of chronic diseases such as hypertension (Mcleod et al., 2019). In particular, power training (PT) has been recommended to improve muscular strength, power and functionality in older adults as it could achieve a greater improvement in functional performance when compared to traditional resistance training (Bottaro et al., 2007). Good levels of functional capacity in older adults are related to a better quality of life, reducing the increase risk of falling, fractures, and hospitalization (Cadore et al., 2013). The characteristics of PT exercises (i.e., low volume and low intensity per set; Chodzko-Zajko et al., 2009) could also attenuate blood pressure (BP) and rate-pressure product responses during exercise (Schimitt et al., 2020), reducing the risk of acute adverse events among individuals with hypertension.

Arterial blood pressure increases during exercise but is depressed after the completion of exercise (Ferrari et al., 2020). This so-called post-exercise hypotension (PEH) is believed to play a major role in BP management as the magnitude of PEH is associated with chronic reductions in BP induced by regular exercise programs (Wegmann et al., 2018). The initial BP level, as well as intensity/volume/type of exercises, can influence both the magnitude and duration of PEH (Pescatello et al., 2004; Domingues et al., 2020). Physiological mechanisms underlying PEH have been under the investigation. The majority of the available studies indicate that the primary factor driving PEH is the reduction in systemic vascular resistance, but a decrease in cardiac output is pointed out as a contributing factor to PEH in older adults (Brito et al., 2014).

Traditional resistance exercises and high-velocity resistance exercise (i.e., PT) are effective strategies to induce PEH under laboratory conditions (Casonatto et al., 2016; Coelho-Júnior et al., 2017; Domingues et al., 2020; Machado Filho et al., 2020). However, few data have demonstrated that this hypotensive effect is not sustained when BP is assessed throughout long periods and under usual conditions in patients with essential hypertension (Queiroz et al., 2015; Oliveira-Dantas et al., 2020; Schimitt et al., 2020). Additionally, a PT session seems to increase the bioavailability of nitric oxide in older women (Coelho-Júnior et al., 2017; Orsano et al., 2018), but studies assessing mechanisms related to PEH after PT in older men are lacking and warrant further investigation. In fact, sex differences exist in the prevalence, awareness, therapy, and prognosis of hypertension and underlying mechanisms responsible for hypertension (Song et al., 2020). The sex influence on PEH was recently described in half-marathon runners (Mourot et al., 2020), attributed to different hemodynamic mechanisms associated with PEH. More specifically, PEH appears to be induced by a reduction in cardiac output in men (Huxley, 2007) whereas a decrease in systemic vascular resistance seems to be a driving factor in women (Parker et al., 2007). TKO

Currently, studies evaluating PT exercise on PEH are scarce and its efficacy remains controversial (Oliveira-Dantas et al., 2020; Schimitt et al., 2020). We have reported no statistically significant differences between PT and Con on ambulatory BP, but a trend toward reduction in daytime (*p* = 0.063) and nighttime (*p* = 0.062) diastolic BP was found after the PT session (Schimitt et al., 2020). Based on this result and considering the potential biological differences between men and women that seems to impact BP responses (Maranon and Reckelhoff, 2013), we decided to run an exploratory analysis, assessing the office and ambulatory BP data separated by sex, and comparing possible differences between older men and women. Additionally, studies comparing acute BP between older men and women are absent, and important sex differences in hemodynamic responses after a single bout of resistance exercise were found in middle-aged adults with normal BP (Mariano et al., 2019). Accordingly, the aims of this secondary, exploratory analysis were to compare the effects of a single bout of PT on PEH, BP variability, and endothelial function between older men and women with hypertension.

## Materials and Methods

### Study Design and Participants

This is an exploratory sub-study of a previously published randomized clinical trial with crossover design (Schimitt et al., 2020) and was conducted in order to compare the sex differences in PEH and its mechanisms after a PT session in older adults. Men and women aged 60 to 75 years with previously diagnosed hypertension by a physician were recruited. Exclusion criteria included previous diagnosis of ischemic heart disease, heart failure, current smokers or ex-smokers for less than 6 months, body mass index over 39.9 kg/m^2^, musculoskeletal problems that restrained them from exercising, changes in antihypertensive medications throughout the trial, diabetes with retinopathy, and participation in structured exercise programs in the last 3 months.

The study was conducted from June 12, 2018, to July 20, 2019, at a tertiary referral hospital in southern Brazil. All participants read and signed an informed consent form before beginning the study. Participation was voluntary, and all ethical principles of confidentiality and data protection were followed. The study protocol was conducted according to the principles of the Declaration of Helsinki and in compliance with the Brazilian legal and regulatory framework for research involving human beings (NR 466/12). The study protocol was approved by the Institutional Review Board of Hospital de Clínicas de Porto Alegre, Brazil, and registered on clinicaltrials.gov under identifier number NCT03615625. The protocol followed the CONSORT guidelines for non-pharmacological treatment (Boutron et al., 2017).

### Preliminary Sessions

Each participant completed a clinical screening and underwent electrocardiogram, BP and heart rate measurements with oscillometric monitor, and anthropometric evaluation in the research laboratory as previously described (Schimitt et al., 2020). The rate-pressure product was calculated using the systolic BP and heart rate values (systolic blood pressure × heart rate). Since most participants included in the study had no previous experience with power exercise training, we implemented two familiarization sessions to ensure that participants perform the prescribed exercises properly. Participants were familiarized with power exercises involved in PT and maximal strength testing during the first two sessions. During the third preliminary session, participant’s maximal strength was evaluated using one repetition maximal strength test (1-RM) in 5 resistance exercises: leg press, bench press, knee extension, upright row, and knee flexion. A specific warm-up composed of 2 sets of 10 and 5 repetitions, using 50 and 75% of estimated 1-RM load was performed prior to the test. After the first attempt, the load was adjusted through Lombardi coefficients, if necessary. Each participant’s 1RM was determined with no more than three attempts with a five-minute recovery between attempts and a two-minute recovery between exercises. These results were used to determine the intensity or load of the experimental sessions. The same trained investigator conducted the tests. Before the test, resting BP was assessed after 20 min of rest in supine position, and these values were used to describe the baseline characteristics of participants.

### Experimental Sessions

The participants performed two experimental sessions in a random order: a power training session (PT) and a non-exercising control session (Con). An epidemiologist generated the randomization list composed of random block sizes of four participants using a computer software. This epidemiologist did not participate in the recruitment or assignment to the experimental sessions. The participants and the research team were blinded to the randomization list until the moment of assignment. Participants were instructed to avoid physical exercise for 24 h before the experimental sessions, keep their usual dietary intake, avoid drinking alcohol and coffee before the experimental sessions, and have the same meal 4 h before each session.

Both experimental sessions started between 2 and 3 PM (at the same time of the day to account for potential diurnal variation in BP and residual effects of antihypertensive medications) and lasted approximately 2 h. A washout period of 5–10 days was implemented between the sessions. Each session was composed of 20 min of rest in the supine position, 40 min of PT or Con protocols followed by 60 min of rest in supine position. Standardized office BP and endothelium-dependent brachial vascular function were assessed before and during the first hour (in intervals of 15 min: post 15', post 30', post 45', and post 60') after exercise and control sessions. Afterwards, participants underwent 24 h ambulatory BP monitoring.

The PT protocol was composed of 3 sets of 10 repetitions of five exercises performed in the following order: leg press, bench press, knee extension, upright row, and knee flexion, using an intensity corresponding to 50% of 1-RM and 2-min intervals between sets and exercises. The concentric phase of exercises during each repetition was performed “as fast as possible,” while the eccentric phase lasted 1 to 2 s. During Con, the participants remained at seated rest on the same exercise equipment, but without any exercise.

### Assessments

Office systolic and diastolic BP was assessed under laboratory conditions using an automatic oscillometric device (Dinamap 1846 SX/P; Critikon, FL, United States; Whincup et al., 1992) according to the Brazilian Guideline of Arterial Hypertension (Malachias et al., 2016). Participants were instructed to remain in supine position and in silence without using any electronic device (i.e., smartphones, notebooks). The use of a supine position to data collection is because BP assessment was performed simultaneously to the flow-mediated dilation (FMD) measurement. A properly-sized cuff was placed on the arm about 2 cm above the antecubital fossa. BP was measured on both arms with a 1-min interval between measures. The arm with the highest B*p* value was used in the characterization, as well as in the pre-and post-intervention evaluation, and the mean BP was calculated automatically by Dinamap.

The 24-h systolic and diastolic BP was assessed through ambulatory BP monitoring (90,702, Spacelabs Medical, WA, United States) after each experiment session in men and women. BP measurements were taken every 15 min at daytime and every 20 min at nighttime. The first daytime period started between 4 and 5 PM (immediately after laboratory session), nighttime between 11 PM and 7 AM, and the second daytime finished at 5 PM on the day after the experimental session. Participants filled a diary about physical activities, symptoms, sleep, and wake-up time. Each examination was considered valid when at least 70% of the expected readings were available and recorded (O’Brien et al., 2013), and the mean BP was calculated automatically by Spacelabs.

Consecutive reading-to-reading BP measurements assessed during ambulatory monitoring were included in a computer software to calculate the average real variability (ARV) (Farrag et al., 2019). ARV weighted for the time interval between consecutive readings was also calculated for both systolic and diastolic BP within the daytime, nighttime, and 24-h periods (Coccina et al., 2019).

The endothelium-dependent brachial vascular function was evaluated using the FMD technique according to guideline (Corretti et al., 2002). Participants were instructed to remain in supine position, and then a BP cuff was placed on the proximal third of the arm (5cm from the cubital fossa). Baseline longitudinal brachial artery diameters were measured along with pulsed doppler signals for flow velocity analysis. After baseline recordings were completed, reactive hyperemia was induced by inflation of a BP cuff to 50mmHg above previously measured systolic BP, and cuff inflation was maintained for 5 min. Two-dimensional images of the brachial artery were acquired using a linear-array multi-frequency transducer (7–12MHz) connected to a high-resolution ultrasound system (HD7XE, Phillips, United States). The time of each image acquisition during the cardiac cycle was determined from simultaneous ECG recording. During image acquisition, anatomical landmarks such as veins and fascial planes were observed to help maintain the same image of the artery throughout the study. The longitudinal image of the brachial artery was recorded continuously for 30 s before (baseline image) to 2 min after cuff deflation (peak diameter). FMD was expressed as the percentage change in arterial diameter from baseline: FMD (%)=(peak diameter − baseline diameter)/baseline diameter × 100. All image analyses were performed offline using the computer software (Brachial Analyzer, Vascular Tools, Medical Imaging Applications, Coralville, IA United States) by an expert evaluator blinded to the sequence of interventions (DeVan et al., 2011).

### Sample Size and Statistical Analyses

This article presents a secondary exploratory analyses, and the original sample size was estimated based on the original hypothesis, considering an initial sample size of 24 individuals with hypertension (12 women and 12 men), able to detect a difference of 5 mmHg in 24-h systolic BP among protocols with 80% of statistical power and a type I error rate of 5%, allowing a dropout rate of up to 10% (Schimitt et al., 2020). Data were entered in duplicate by three different researchers. The statistician did not participate in the recruitment or assignment to the experimental sessions and was blinded to the interventions. The assumption of normality was evaluated through the use of the Shapiro–Wilk test. Results were expressed as means and standard deviation (Table 1) or standard error (Tables 2, 3, and 4) for variables with normal distribution. In office BP data, average total 1 h was calculated using the average of the 4 values measured after the sessions ((post 15'+ post 30' + post 45' + post 60')/4). The post-FMD values were calculated using the average of 2 consecutive values measured after the sessions (post media 30' = (post 15' + post 30')/2; post media 60' = (post 45' + post 60')/2). Delta office BP was calculated through the difference between PT and Con sessions in each time-point after intervention (i.e., post 15', post 30', post 45', post 60, and average post 1 h), adjusted for baseline (pre) BP values [(post-exercise BP–pre-exercise BP)–(post-control BP–pre-control BP)] in men and women.

**Table 1:** Selected participant characteristics.

	Men (*n* = 12)	Woman (*n* = 12)	*p* value
Age, years (±SD)	67 (5)	67 (4)	0.785
Race/Ethnicity, n (%)			0.237
White	12 (100)	9 (75)	
Black	0 (0.0)	2 (17)	
Asian	0 (0.0)	1 (8)	
*Anthropometric measures*			
Body weight, kg (range)	92 (79–100)	70 (66–74)	**0.001**
Height, cm (range)	171 (164–174)	157 (155–159)	**<0.001**
BMI, kg/m^2^ (SD)	31 (4)	28.2 (3)	**0.047**
Waist circumference, cm (SD)	107 (13)	92 (9)	**0.003**
Anti-hypertensive medications, n (range)	2 (1–4)	2 (1–4)	
Diuretics, n (%)	9 (75)	10 (83)	0.721
β blockers, n (%)	3 (25)	4 (33)	0.423
ACE inhibitors, n (%)	4 (33)	4 (33)	0.833
Angiotensin receptor antagonists, n (%)	9 (75)	5 (42)	0.374
Calcium channel blockers, n (%)	1 (8)	4 (33)	0.174
*Hemodynamic measures (± SD)*			
Systolic BP, (mmHg)	133 (12)	133 (14)	0.964
Diastolic BP (mmHg)	76 (9)	76 (8)	0.846
Mean BP, (mmHg)	96 (8)	98 (8)	0.567
Heart rate, (bpm)	65 (12)	68 (11)	0.634
RPP, (mmHg^*^bpm)	8,661 (1721)	8,987 (1796)	0.654
*1-RM muscle strength tests, Kg (± SD)*			
Leg Press	170.2 (61)	105.0 (27)	**0.003**
Knee Extension	103.3 (29)	69.8 (14)	**0.003**
Knee Flexion	65.9 (17)	41.1 (10)	**0.001**
Bench Press	50.0 (11)	25.9 (6)	**<0.001**
Upright row	38.2 (17)	21.4 (5)	**<0.001**
Total load lifted (kg)	427.6 (121)	263.3 (53)	**<0.001**
Total load/body weight	4.6 (0.9)	3.8 (0.7)	**0.011**

Values are means ± standard derivation (SD) for parametric data, and medians ± interquartile interval (range) for nonparametric data; BMI, body mass index; ACE, angiotensin-converting enzyme; BP, blood pressure; RPP, rate-pressure product; 1-RM, repetition maximal strength. Total load lifted: sum of the 1RM test values.

**Table 2:** Office blood pressure measures before (pre) and after (for 1 h) the power training and control sessions in men and women.

Variables	Control	Power training	Intervention		Sex	
ΔBP (mmHg)	*p* value	ΔBP (mmHg)	*p* value
*Systolic* *Pre*						
Men	130.4 ± 4.0	129.0 ± 3.5	−1.4 ± 2.9	0.622	3.6 ± 5.3	0.191
Women	134.5 ± 3.5	129.5 ± 4.4	−5.0 ± 3.8	0.191		
*Post 15'*						
Men	136.6 ± 4.6	131.7 ± 5.1	−4.9 ± 3.4	0.151	5.4 ± 7.3	0.437
Women	142.4 ± 5.9	132.1 ± 5.2	−10.3 ± 2.5	**<0.001**		
*Post 30'*						
Men	141.5 ± 5.4	132.2 ± 4.6	−9.3 ± 2.9	**0.001**	−3.3 ± 6.4	0.400
Women	143.6 ± 5.1	137.6 ± 4.5	−6.0 ± 3.4	0.074		
*Post 45'*						
Men	145.1 ± 5.5	122.1 ± 11.3	−23.0 ± 10.7	**0.031**	−16.9 ± 12.7	0.103
Women	148.9 ± 4.9	142.8 ± 5.7	−6.1 ± 4.5	0.178		
*Post 60'*						
Men	150.0 ± 5.4	131.3 ± 4.5	−18.7 ± 3.6	**<0.001**	−15.0 ± 7.2	**0.048**
Women	149.1 ± 4.8	145.5 ± 5.6	−3.7 ± 3.3	0.260		
*Average post 1 h*						
Men	143.3 ± 4.9	129.3 ± 5.7	−13.9 ± 4.4	**<0.001**	−7.4 ± 4.9	**0.046**
Women	146.0 ± 5.0	139.5 ± 4.9	−6.5 ± 2.8	**0.046**		
*Diastolic* *Pre*						
Men	76.4 ± 3.1	75.3 ± 2.1	−1.1 ± 1.7	0.516	0.7 ± 3.5	0.491
Women	74.6 ± 2.9	72.8 ± 2.9	−1.8 ± 2.9	0.554		
*Post 15'*						
Men	80.2 ± 3.5	74.7 ± 3.0	−5.5 ± 2.7	**0.043**	−2.6 ± 4.3	0.537
Women	77.3 ± 3.1	74.4 ± 3.1	−2.9 ± 2.3	0.202		
*Post 30'*						
Men	81.8 ± 3.4	74.7 ± 2.9	−7.1 ± 2.4	**0.003**	−6.6 ± 4.7	0.079
Women	77.8 ± 3.3	77.3 ± 2.8	−0.5 ± 2.4	0.861		
*Post 45'*						
Men	82.6 ± 3.4	76.2 ± 3.3	−6.5 ± 2.3	**0.004**	−5.0 ± 4.5	0.352
Women	78.4 ± 3.0	76.9 ± 3.1	−1.5 ± 2.9	0.601		
*Post 60'*						
Men	87.0 ± 3.5	76.0 ± 2.8	−11.0 ± 1.7	**0.001**	−9.2 ± 4.9	**0.049**
Women	78.4 ± 3.4	79.2 ± 3.1	1.8 ± 2.9	0.752		
*Average post 1 h*						
Men	82.9 ± 3.3	75.4 ± 2.8	−7.5 ± 2.2	**<0.001**	−6.0 ± 2.2	0.052
Women	77.9 ± 3.0	76.9 ± 2.9	−1.0 ± 2.2	0.638		

Values: mean ± SE; BP, blood pressure. *p*-value in the intervention is comparing the interventions separately in men and women (power training vs control). *p*-value in the sex is comparing the results of the sexes in the interventions (men vs women).

**Table 3:** Ambulatory blood pressure measures after the power training and control sessions in men and women.

Variables	Control	Power training	Intervention		Sex	
			ΔBP (mmHg)	p value	ΔBP (mmHg)	p value
***Systolic BP*** *24-h*						
Men	130.8 ± 4.2	130.7 ± 3.4	−0.1 ± 2.7	0.973	−0.3 ± 4.8	0.835
Women	130.9 ± 3.6	131.1 ± 3.4	0.2 ± 1.6	0.915		
*Daytime*						
Men	134.2 ± 4.3	136.1 ± 3.7	1.2 ± 2.5	0.447	−0.2 ± 5.0	0.511
Women	133.2 ± 4.0	134.2 ± 3.4	1.0 ± 1.3	0.451		
*Nighttime*						
Men	122.2 ± 4.9	119.0 ± 4.9	−3.2 ± 2.9	0.272	−1.3 ± 5.4	0.401
Women	125.4 ± 3.7	123.5 ± 3.6	−1.9 ± 3.2	0.543		
***Diastolic BP***						
*24-h*						
Men	77.6 ± 3.2	75.4 ± 3.1	−2.2 ± 1.6	0.178	−2.4 ± 4.1	0.709
Women	76.0 ± 2.6	76.2 ± 2.7	0.2 ± 0.8	0.833		
*Daytime*						
Men	80.3 ± 3.4	79.3 ± 3.0	−1.0 ± 1.3	0.427	−1.8 ± 4.1	0.366
Women	78.7 ± 2.9	79.4 ± 2.7	0.8 ± 0.7	0.309		
*Nighttime*						
Men	69.8 ± 2.9	67.0 ± 3.2	−2.8 ± 2.1	0.180	−1.4 ± 4.2	0.608
Women	70.6 ± 2.2	69.2 ± 2.7	−1.4 ± 1.9	0.457		

Values: mean ± SE; BP, blood pressure (mmHg); Δ: net effect. Intervention: Δ BP and *p*-value corresponding to the individual differences between control and power training sessions in each sex. Sex: Δ BP and P value corresponding to the differences between men and women.

**Table 4:** Exploratory analyses of ambulatory blood pressure, separated by sex, after the power training and control sessions.

Variables	Control	Power training	Intervention	
			ΔBP (mmHg)	*p* value
***Systolic*** *24-h*				
Men	132.5 ± 6.6	129.8 ± 4.5	−2.8 ± 2.9	0.336
Women	129.7 ± 3.0	129.6 ± 2.3	−0.1 ± 1.6	0.943
*Daytime*				
Men	135.5 ± 6.7	135.1 ± 5.2	−0.4 ± 2.6	0.891
Women	131.8 ± 3.4	132.7 ± 2.4	0.9 ± 1.4	0.505
*Nighttime*				
Men	123.7 ± 7.5	117.4 ± 4.9	−6.3 ± 3.1	**0.042**
Women	124.7 ± 3.7	122.1 ± 2.6	−2.6 ± 3.1	0.398
***Diastolic*** *24-h*				
Men	78.1 ± 4.1	74.2 ± 3.1	−3.9 ± 1.9	**0.036**
Women	75.1 ± 1.9	75.1 ± 2.0	0.0 ± 0.8	0.973
*Daytime*				
Men	80.3 ± 4.2	78.1 ± 3.2	−2.2 ± 1.3	0.109
Women	77.6 ± 2.3	78.4 ± 2.0	0.8 ± 0.7	0.244
*Nighttime*				
Men	70.7 ± 3.7	65.5 ± 3.0	−5.2 ± 2.5	**0.036**
Women	69.9 ± 2.1	68.1 ± 2.1	−1.9 ± 1.9	0.319

Values: mean ± SE; BP, blood pressure. *p*-value in the intervention is comparing the interventions separately in men and women (power training vs control).

Generalized estimating equations (GEE) analyses were used to assess main effects between experimental sessions (2 sessions: PT and Con) by time in men and women (session^*^time). To compare the sex differences, we run an additional GEE analysis including a new factor (sex: men *vs.* women), adjusted for BMI, since we found a difference in BMI between men (31 kg.m^−2^) and women (28 kg.m^−2^) that can affect BP values (session^*^ time^*^sex). Post-hoc comparisons were performed using Bonferroni tests. Statistical significance was set *a priori* at *p* < 0.05, and a borderline significance was detected for *p*-values ranging from 0.05 to 0.10. All statistical analyses were performed using SPSS Statistics for Windows version 22.0 (IBM, Armonk, NY, United States).

## Results

**Figure 1:**
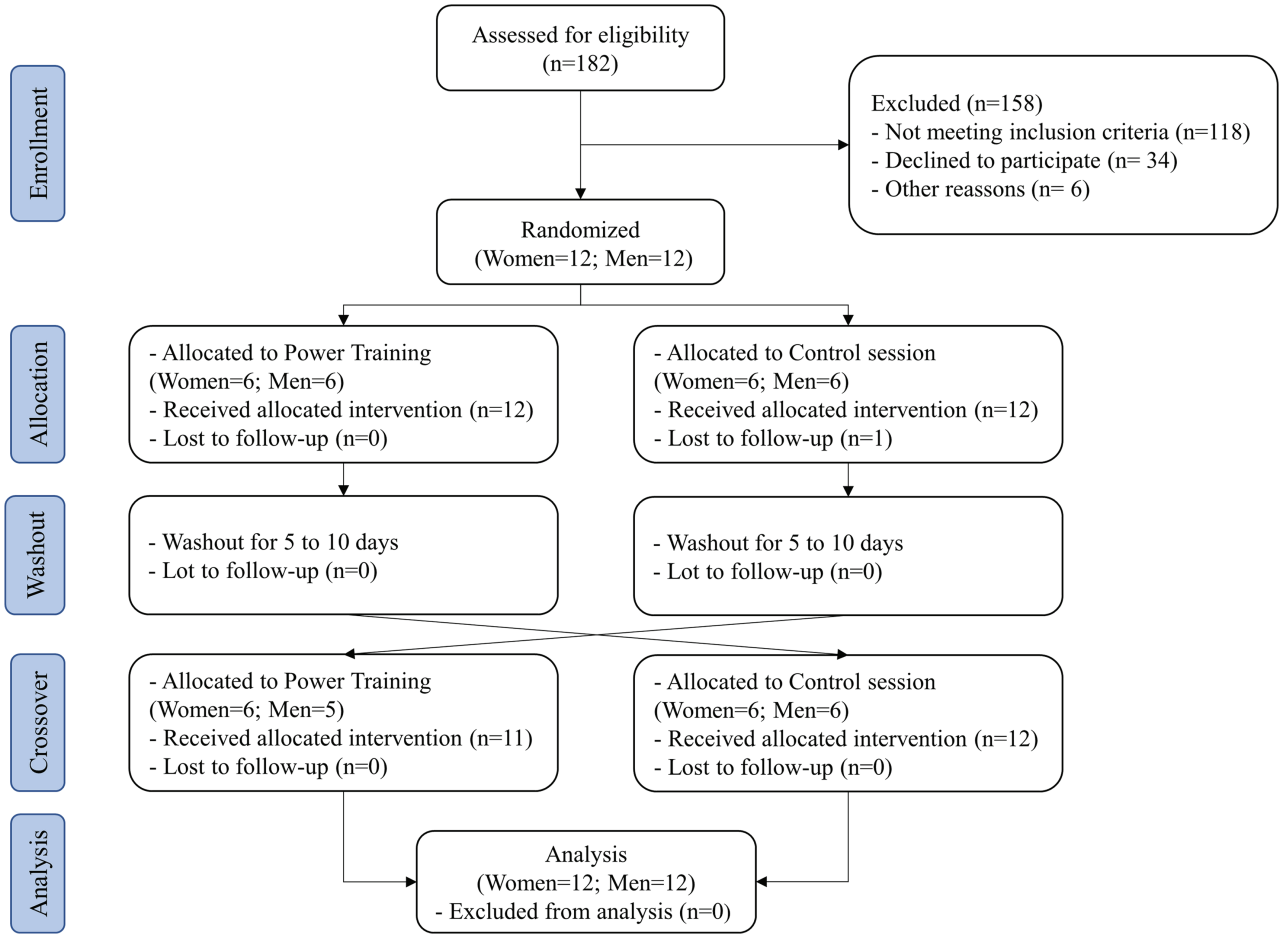
Flow diagram of participants.

A flowchart of the experiments is presented in Figure 1. Participants’ characteristics at baseline, assessed during the preliminary sessions, are shown in Table 1. There were no reported adverse events during the PT or Con session. All participants performed the same amount of exercise during the PT protocol (i.e., 3 × 10 at 50%1RM in 5 resistance exercises). The quality of the ambulatory BP recorded was considered satisfactory in all patients. Additionally, the participants reported no adverse symptoms or difficulties to sleep throughout the study.

Office BP data are presented in Table 2. Time^*^session^*^sex interaction was found in office BP for systolic BP (*p* < 0.001) and diastolic BP (*p* < 0.001). The BP values before the PT and Con sessions were similar, and no difference between men and women was found at baseline (*p* >0.05). Comparing the BP values in the first hour after each experimental session with the corresponding baseline BP, no significant change was found after PT, while an increased BP after Con was found (*p* < 0.05). Comparing PT with Con, a reduced systolic BP was found at post 30' (*p* = 0.001), post 45' (*p* = 0.031), post 60' (p < 0.001), and average total 1 h (p < 0.001) in men, and at post 15' (p < 0.001) and average total 1 h (*p* = 0.046) in women. Additionally, a reduced diastolic BP was found after PT when compared with Con at post 15' (*p* = 0.043), post 30' (*p* = 0.003), post 45' (*p* = 0.004), post 60' (*p* = 0.001), and average total 1 h (p < 0.001) only in men. Comparing BP response after the first hour between men and women, a reduced systolic BP at post 60' (*p* = 0.048) and average total 1 h (*p* = 0.046), and a reduced diastolic BP at post 60' (*p* = 0.049) were found in men.

Ambulatory BP data are presented in Tables 3 and 4. No time^*^session^*^sex interaction was found in systolic (*p* = 0.335) and diastolic (*p* = 0.208) ambulatory BP (Table 3). Additionally, we run an exploratory analysis to assess the main effects between PT and Con in men and women separately. Time^*^session interaction was found in ambulatory BP for systolic (*p =* 0.041), and a borderline time^*^session interaction was found for diastolic BP (*p* = 0.050) in men but not in women. Compared with Con, 24-h diastolic BP decreased after PT in men. Nighttime systolic and diastolic BP also decreased after PT in men. In women, no statistically significant differences were found between PT and Con sessions for daytime, nighttime, and 24-h systolic/diastolic BP (Table 3).

BP variability and FMD data are presented in Table 5. No time^*^session^*^sex interaction was found for BP variability and FMD data.

**Table 5:** Short-term blood pressure variability and endothelial function measures after the power training and control sessions in men and women.

Variables	Control	Power training	Intervention		Sex	
			ΔBP (mmHg)	p value	ΔBP (mmHg)	*p* value
*Systolic BP variability* *24-h*						
Men	8.6 ± 0.4	8.4 ± 0.4	0.2 ± 0.4	0.240	0.4 ± 0.6	0.351
Women	8.8 ± 0.3	8.9 ± 0.4	0.2 ± 0.4	0.618		
*Daytime*						
Men	9.2 ± 0.6	8.6 ± 0.4	0.7 ± 0.6	0.077	−1.0 ± 0.6	0.106
Women	9.2 ± 0.4	9.5 ± 0.4	0.3 ± 0.5	0.589		
*Nighttime*						
Men	7.7 ± 0.4	7.9 ± 0.5	0.2 ± 0.3	0.839	0.3 ± 0.8	0.718
Women	8.2 ± 0.6	8.1 ± 0.6	0.1 ± 1.0	0.928		
*Diastolic BP variability* *24-h*						
Men	6.9 ± 0.3	6.6 ± 0.3	0.3 ± 0.4	0.457	0.2 ± 0.6	0.667
Women	6.9 ± 0.5	6.9 ± 0.5	0.1 ± 0.5	0.827		
*Daytime*						
Men	7.1 ± 0.3	6.5 ± 0.4	0.6 ± 0.3	0.062	0.3 ± 0.7	0.409
Women	7.4 ± 0.5	7.1 ± 0.6	0.3 ± 0.5	0.478		
*Nighttime*						
Men	6.9 ± 0.4	7.1 ± 0.6	0.3 ± 0.7	0.591	0.4 ± 0.8	0.681
Women	6.1 ± 0.7	6.8 ± 0.5	0.7 ± 0.7	0.398		
*Flow-mediated dilatation (%)* *Pre*						
Men	4.8 ± 0.3	4.9 ± 0.3	0.1 ± 0.2	0.564	0.8 ± 1.2	0.575
Women	6.8 ± 0.8	6.1 ± 1.4	0.7 ± 1.1	0.515		
*Post media 30'*						
Men	4.6 ± 0.5	5.1 ± 0.3	0.5 ± 0.6	0.374	0.3 ± 1.1	0.517
Women	6.3 ± 0.7	6.5 ± 0.7	0.2 ± 0.8	0.778		
*Post media 60'*						
Men	5.7 ± 0.3	5.0 ± 0.3	0.7 ± 0.5	0.153	1.7 ± 1.7	0.825
Women	5.8 ± 0.5	6.8 ± 1.5	1.0 ± 1.0	0.354		

Values: mean ± SE; BP, blood pressure (mmHg); ARV, average real variability (mmHg); Δ: net effect. Intervention: Δ BP and *p*-value corresponding to the individual differences between control and power training sessions in each sex. Sex: Δ BP and *p*-value corresponding to the differences between men and women.

## Discussion

To the best of our knowledge, no previous studies have evaluated sex differences in acute blood pressure and hemodynamic responses after the PT session in older adults with essential hypertension. A salient finding of this exploratory study was a reduced office BP values after exercise in men than women, highlighting important differences between older men and women on PEH after the PT protocol. Additionally, no significant difference between men and women was found in ambulatory BP, endothelial function as assessed by FMD, and short-term BP variability. Given the limited sample size of the present study, further studies are necessary to understand how PEH is mediated in older hypertensive individuals. The differences in BP responses to PT between older men and women are highlighted and deserve further studies to deepen the physiological mechanisms associated with PEH.

Scarcity data have suggested that men and women presented similar BP responses after a single bout of resistance training (Senitko et al., 2002; Queiroz et al., 2013). Controversially, a recent study assessing PEH after a traditional resistance exercise protocol in middle-aged men and women with normal BP found BP reduction in men but not in women (Mariano et al., 2019). In the present study, we specifically explore differences between older men and women using office and ambulatory BP data. To the best of our knowledge, this is the first study to compare older men and women with hypertension in PEH induced by PT. We found differences between older men and women on PEH after PT in office BP. Systolic and diastolic BP was lower in men than women after the first hour (post 60’ systolic BP: –15 mmHg, *p* = 0.048; Post 60’ diastolic BP: –9 mmHg, *p* = 0,049). The magnitude and duration of PEH seem to be influenced by intensity/volume of exercise (Pescatello et al., 2004; Domingues et al., 2020), and differences in total overload (sets × repetitions × total load lifted × number of exercises x time under tension) can be a key component in predicting the magnitude and duration of PEH after different resistance exercise protocols. In our study, the relative amount of exercise performed during the PT protocol was identical between men and women (3 × 8–10 - 50%1RM - 5 exercises). However, men performed higher total overload than women due to their greater values of maximal strength (i.e., 1RM tests; Table 1). We believe that the total overload performed during the PT session (i.e., sets × repetitions × number of exercises × total load lifted × time under tension) seems to be the main factor that helps to explain the above-mentioned differences between men and women, influencing the magnitude and duration of PEH after PT in older adults.

Ambulatory BP monitoring is the best strategy to observe and understand the BP behavior throughout daily living activities and sleeping periods (Grossman, 2013). Only two studies have evaluated ambulatory BP after a single bout of PT in older adults with hypertension (Oliveira-Dantas et al., 2020; Schimitt et al., 2020). In the study of Oliveira-Dantas et al. (2020), a PT protocol (i.e., three sets of six repetitions in eight exercises using elastic bands) was performed by 14 hypertensive older women and no significant difference between PT and Con in 24-h, daytime, and nighttime BP was found. In the present study, no statistical differences in men and women were found in daytime, nighttime, and 24-h BP when using the three factors in the same GEE analysis (session^*^time^*^sex). Additionally, we run an exploratory analysis (Table 4), splitting our sample and assessing the ambulatory BP data separated by sex (session^*^time), and found significant reduction in 24-h diastolic BP after PT when compared with Con in older men but not in women, as well as nighttime ambulatory BP reduction only in men. We assumed that the inclusion of an extra factor in our analysis, using a limited sample size, reduced the statistical power of the study. Based on the limitation of this exploratory analysis, future studies should include a larger sample size to confirm the present results.

In an attempt to provide insight into physiological mechanisms, we assessed endothelium-dependent vasodilation using FMD, and short-term BP variability as an index of cardiovagal baroreflex sensitivity (Floras et al., 1988). The improvement in endothelial function after exercising is one of the potential mechanisms that could help explaining the occurrence of PEH (Halliwill et al., 2013). We found no significant difference in endothelial function between the PT and Con sessions. Additionally, changes in blood pressure were not associated with the corresponding changes in FMD. Similar results have been reported in previous studies that also evaluated FMD at different times after exercise sessions as they did not observe decreases in this variable (Birk et al., 2013; Katayama et al., 2013). Short-term BP variability has an important association with autonomic cardiovascular system, and higher BP fluctuations can be associated to impairments on autonomic nervous system that results in high BP levels (Nardin et al., 2019). Few studies have evaluated the acute effects of exercise on ambulatory BP variability (Caminiti et al., 2019; Batista et al., 2020; Matias et al., 2020), and no previous studies have compared BP variability between older men and women after a single bout of exercise. A previous study demonstrated reduction in 24 h and daytime systolic (~2 mmHg) and diastolic (~1 mmHg) BP variability after a single bout of combined aerobic and resistance exercise in postmenopausal women with hypertension (Matias et al., 2020). However, the present study did not find reductions on ambulatory BP variability after PT. Further studies are necessary to deepen the physiological mechanisms associated with PEH.

Some limitations of the present study should be taken into account in order to properly interpret the present results. The limited number of participants enrolled in this study is due to the hypothesis tested in the original trial (Schimitt et al., 2020). However, most of the previous data assessing PEH have also used similar sample sizes. Clearly, a larger sample size trial is mandatory to confirm the present results. The endothelium-dependent brachial vascular function assessment using the FMD technique and recording data throughout 2 min after releasing the cuff could also be considered a limitation, since a recent guideline suggests that time to peak dilation in sedentary older adults can occur after the 2-min period (Thijssen et al., 2019). The enrollment of untrained participants aged 60–75 years may have limited the generalization of our findings to younger adults or trained older adults. However, the analyses of well-functioning and untrained older adults are more likely to represent the elderly population. Our study provides important implications for the exercise prescription targeted to aging individuals with essential hypertension. The use of a resistance exercise protocol is highly recommended to older adults (Fragala et al., 2019), exploring potential sex differences on PEH that should be taken into account in order to prescribe PT for hypertensive older adults. Additionally, considering that the magnitude of PEH after exercise is directly related to the baseline BP (Queiroz et al., 2015), it is reasonable to assume that the PT protocol could be more effective in reducing BP in participants with uncontrolled hypertension. The use of researchers blinded to interventions for outcome assessment and analysis and the BP assessment using ambulatory BP monitoring, the gold-standard method to assess PEH, are also strengths of this study.

## Conclusion

In older adults with hypertension, the office BP response after the experimental sessions was different in men and women, showing that the PT protocol is more effective to acutely reduce BP in men. Additionally, the mechanisms behind this result remain unclear. Moreover, the exploratory results for ambulatory BP suggest the need of further studies to assess the potential difference between men and women.

This finding highlights the relevance of this type of resistance training as a non-pharmacological strategy to acutely reduce BP in older men with hypertension and reduced physical capacity. In women, however, the duration of PEH after our PT protocol was very limited. Different PT protocols might be necessary to induce benefits among women. Our findings have important implications for exercise prescription targeting older individuals with hypertension in that sex cannot be combined to analyze PEH.

## Data Availability Statement

The raw data supporting the conclusions of this article will be made available by the authors, without undue reservation.

## Ethics Statement

The studies involving human participants were reviewed and approved by Institutional Review Board of Hospital de Clínicas de Porto Alegre, Brazil. The patients/participants provided their written informed consent to participate in this study.

## Author Contributions

LC: conceptualization, investigation, and writing—original draft. RS and LD: investigation, formal analysis, and writing—review and editing. TA, HT, and SF: formal analysis and writing—review and editing. RF: conceptualization, investigation, formal analysis, funding acquisition, writing—original draft, and writing—review and editing. All authors contributed to the article and approved the submitted version.

### Conflict of Interest

The authors declare that the research was conducted in the absence of any commercial or financial relationships that could be construed as a potential conflict of interest.
